# The management of acute parathyroid crisis secondary to parathyroid carcinoma: a case report

**DOI:** 10.1186/1752-1947-4-28

**Published:** 2010-01-29

**Authors:** Kathy Rock, Nariman Fattah, Diarmuid O'Malley, Enda McDermott

**Affiliations:** 1 Surgical Professorial Unit, Saint Vincent's University Hospital, Dublin 4, Ireland

## Abstract

**Introduction:**

Hypercalcaemic hyperparathyroid crisis is a rare but life-threatening complication of primary hyperparathyroidism. Parathyroid carcinoma is a rare malignancy with an incidence of 0.5% to 4% of all reported cases of primary hyperparathyroidism.

**Case presentation:**

We report the case of a 60-year-old Caucasian man with hypercalcaemic hyperparathyroid crisis associated with parathyroid carcinoma. He presented with a classic hypercalcaemic syndrome and his serum calcium and parathyroid hormone levels were at 4.65 mmol/L and 1743 ng/L, respectively. He initially presented with a two-week history of weakness and lethargy and a one-week history of vomiting, polyuria and polydipsia. An emergency left thyroid lobectomy and left lower parathyroidectomy were performed. There was a prompt decrease in his parathyroid hormone level immediately after surgery. Histology revealed that our patient had a 4-cm parathyroid carcinoma.

**Conclusion:**

In patients with parathyroid carcinoma, the optimal surgical treatment is *en bloc *resection with ipsilateral thyroid lobectomy and removal of any enlarged or abnormal lymph nodes. Surgery is the only curative treatment. In our patient, prompt surgical intervention proved successful. At six months the patient is well with no evidence of disease recurrence. This case highlights the importance of considering a hyperparathyroid storm in the context of a parathyroid carcinoma. Parathyroid carcinoma is a rare entity and our knowledge is mainly derived from case reports and retrospective studies. This case report increases awareness of this serious and life-threatening complication. This report also illustrates how prompt and appropriate management provides the best outcome for the patient.

## Introduction

Hypercalcaemic hyperparathyroid crisis is a rare but life-threatening complication of primary hyperparathyroidism. The symptoms of hypercalcaemia are frequently non-specific and reflect multi-organ involvement. This condition should be suspected in acutely ill patients with profound dehydration, gastrointestinal manifestations, urinary symptoms, altered mental state, or cardiac arrhythmias. Parathyroid carcinoma is a rare malignancy with an incidence of 0.5% to 4% of reported cases of primary hyperparathyroidism [1,2].

In patients with parathyroid carcinoma, the optimal surgical treatment is *en bloc *resection with ipsilateral thyroid lobectomy and the removal of any enlarged or abnormal lymph nodes [3]. Surgery is the only curative treatment. The cure rate is reported as being as high as 98% [4].

## Case presentation

**Figure 1:**
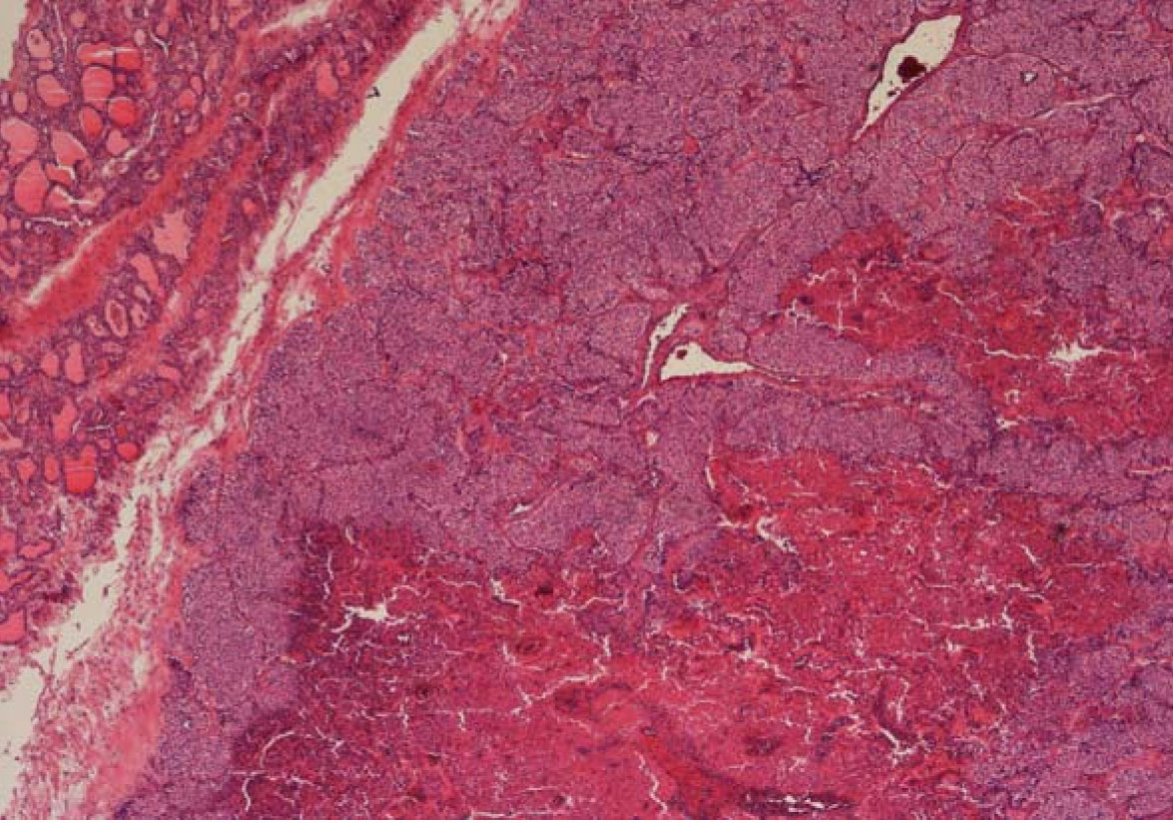
**Parathyroid carcinoma × 4**.

A 60-year-old Caucasian man was transferred from a regional hospital to a tertiary referral centre for the emergency management of hypercalcaemic hyperparathyroid crisis. He initially presented with a two-week history of weakness and lethargy and a one-week history of vomiting, polyuria and polydipsia. He became acutely confused in the 24 hours prior to his admission to our hospital and registered 12 on the Glasgow Coma Scale. On examination he was normotensive with a regular pulse of 70 beats per minute. There was a left-sided mass in the anterior triangle of his neck measuring 3 × 3 cm. The mass was firm, regular, non-tender and mobile. See Figure 1 and Table1 for results of the initial laboratory investigations.

**Table 1:** Results of the initial laboratory investigations

Investigation	Test	Result	Reference range
FBC	HB	10.5 g/l	13-18 g/l
	Haematocrit	0.26	0.4-0.54
	Platelets	388 10 × 9/l	150-400 10 × 9/l
	WCC	23.3	2.20-2.60
Renal function	Urea	29.2 mmol/l	2.1-7.1 mmol/l
	Creatinine	309 μmol/l	62-106 μmol/l
	Troponin	4.64	0.00-0.03
	CK	653 U/l	1-185 U/l
Metabolic profile	PTH	1743 ng/l	12-64 ng/l
	Serum Calcium	4.65 mmol/l	2.20-2.60 mmol/l
	Ionised Calcium	2.16 mmol/l	1.19-1.35 mmol/l
Liver function tests	Alkaline	97 U/l	35-129 U/l
	Phosphatase	57 U/l	8-61 U/l
	GGT	27 g/l	25-50 g/l
	Albumin INR (ratio)	1.05	
Electrolytes	Sodium	133 mmol/l	135-145 mmol/l
	Potassium	3.4 mmol/l	3.5-5.0 mmol/l

An ultrasonography of his neck showed a 4 × 3 cm large cyst in the left lobe of his thyroid gland. His parathyroid glands were not visualised. A 99 mTc-sestamibi scintigraphy scan was performed, and no evidence of a parathyroid adenoma was found. An electrocardiogram revealed acute changes with ST depression in leads II, III, aVF and V2 to V6. An echocardiogram showed good left ventricular failure (LVF) with an ejection fraction of 63%.

Initial management included aggressive fluid resuscitation, cardiac monitoring and the administration of intravenous bisphosphonates. A left thyroid lobectomy and left lower parathyroidectomy were performed. At the time of operation, a haemorrhagic cyst with a parathyroid gland within it was visualised. A biopsy was taken from the left upper parathyroid gland. Histology revealed a 4 cm parathyroid carcinoma within the cyst which was fully excised. The cyst had a macroscopic measurement of 6 × 6 × 5 cm. The wall of the cyst contained a well-circumscribed, unencapsulated soft tissue mass measuring 4 × 2.5 × 0.7 cm. It was light yellow-tan in colour and firm in consistency. There was a small amount of normal thyroid parenchyma within the specimen. The biopsy of the left upper parathyroid gland revealed normal parathyroid parenchyma without diagnostic abnormality.

**Figure 2:**
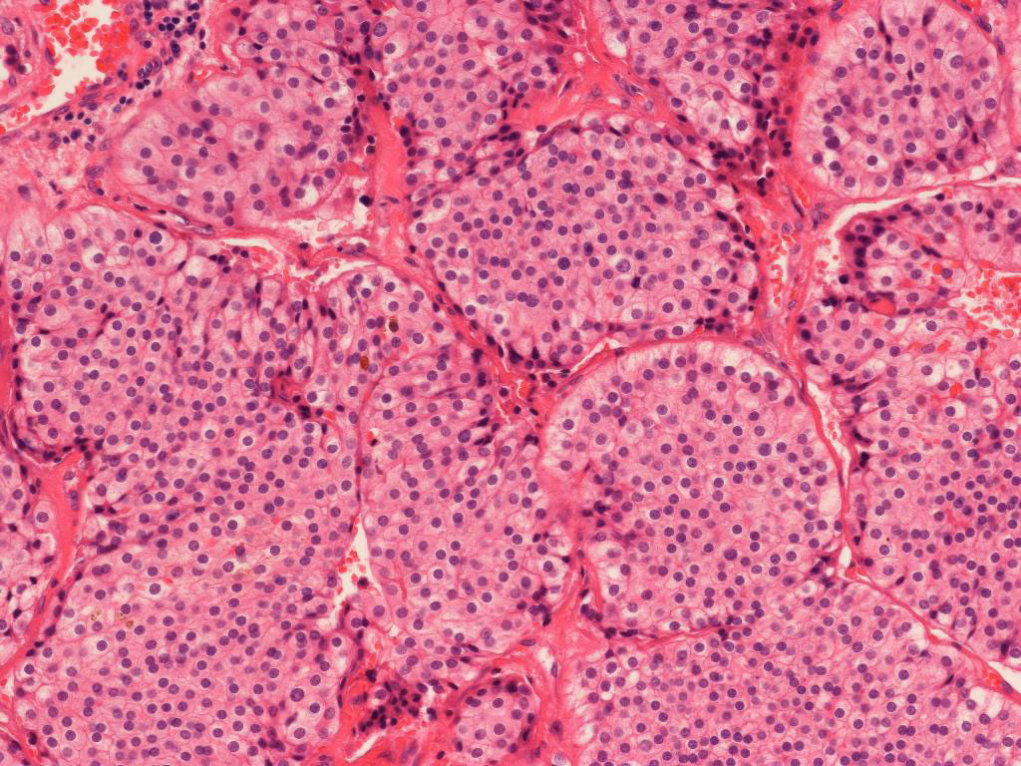
**Parathyroid carcinoma × 20**.

**Figure 3:**
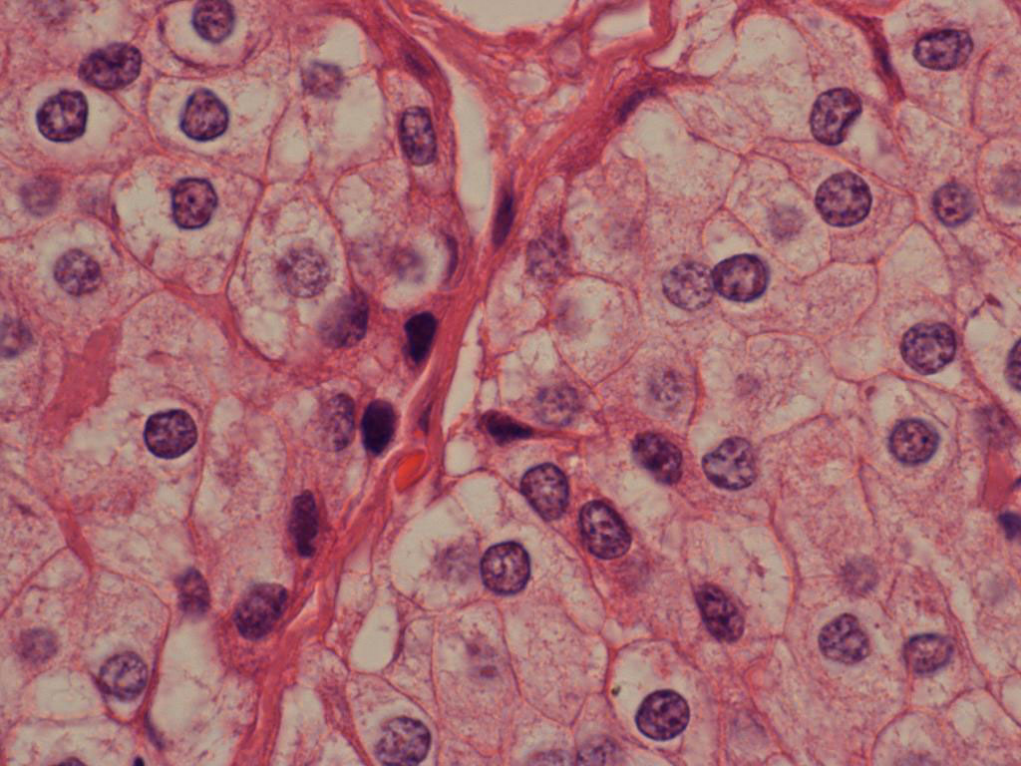
**Parathyroid carcinoma × 100**.

**Figure 4:**
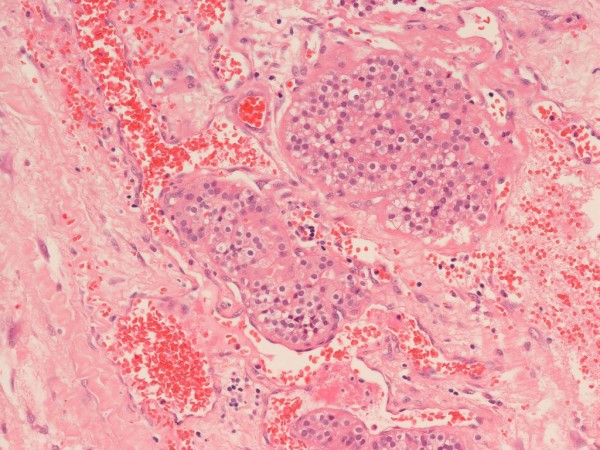
**Parathyroid carcinoma lymphovascular invasion**.

Our patient remained intubated and ventilated overnight in the intensive care unit. His metabolic laboratory profile resolved quickly following the surgery [Figure 2, Figure 3, Figure 4]. A lower respiratory tract infection delayed his initial recovery. He was discharged home on oral calcium supplementation 24 days after surgery. He has been followed up for 6 months so far without any complications or disease recurrence.

## Discussion

In 1850, Sir Richard Owen was the first to describe the parathyroid glands after performing an autopsy of an Indian rhinoceros. The first parathyroid surgery was undertaken by Felix Mandl in Vienna in 1925.

Parathyroid carcinoma is a rare malignancy with an incidence of 0.5% to 4% in patients with primary hyperparathyroidism. The clinical presentation of parathyroid carcinomas and adenomas can be very similar, although patients with parathyroid carcinoma register significantly higher serum calcium, parathyroid hormone (PTH) and alkaline phosphatase levels compared with patients with adenomas (see Table2). A palpable neck mass has been reported in 30% to 76% of patients with parathyroid carcinoma. This important clinical finding constitutes a difference between benign and malignant parathyroid diseases, as a palpable neck mass is distinctly unusual in primary hyperparathyroidism [5]. In addition, recurrent laryngeal nerve palsy in a patient with primary hyperparathyroidism who has not had any previous neck surgery is also very suggestive of parathyroid cancer.

**Table 2:** Preoperative and postoperative courses of serum calcium, ionized calcium and parathyroid hormone

	ON ADMISSION	DAY 1 POST OP	DAY 4 POST OP
Corrected serum calcium (ref: 2.20-2.60 mmol/L)	4.65	3.55	3.28
Ionised calcium (ref: 1.19-1.35 mmol/L)	2.43	2.16	2.11
Parathyroid Hormone (ref: 12-64 ng/L)	1743	59.3	31.1

Our knowledge of this rare condition is based on case reports and retrospective studies. One centre affiliated to the University of Crete reported five cases of sporadic parathyroid carcinoma over a 12-year period. The clinical presentation of the disease in these five patients was varied. The imaging modalities utilised in these patients were ultrasonography, Sestamibi scan and computed tomography (CT), all of which were able to locate parathyroid carcinoma in the patients. Four lesions were localized using ultrasonography and Sestamibi scan and one by CT. This case series highlighted the need for a high index of clinical suspicion to allow for prompt surgical intervention. It also highlighted the importance of initial surgical management, as prognosis can depend on the success of the first operation. It is also important to remember the serious malignant potential of this lesion at the time of resection [6].

There are many different modalities for the preoperative localization of the parathyroid lesions. In our patient, a Sestamibi scan was preformed. In several centres, CT is preformed in patients with parathyroidism as it allows for improved preoperative planning for directed parathyroidectomy with a four-dimensional CT. CT is able to depict hyperfunctioning glands and to search for metastasis and abnormal lymph glands. Four-dimensional CT is a unique tool that allows for a precise preoperative localization of hyperfunctioning parathyroid glands [7].

In cases of primary hyperthyroidism, surgical exploration of the neck and resection of the hyperfunctioning parathyroid tissue should be undertaken without further delay (within 72 hours). Parathyroidectomy by an experienced surgeon usually has a low complication rate (2.3%) and results in the patient's prompt recovery. The cure rate is reported as being as high as 98% [8].

In patients with parathyroid carcinoma, the optimal surgical treatment is *en bloc *resection with ipsilateral thyroid lobectomy and the removal of any enlarged or abnormal lymph nodes. Initial appropriate and aggressive resection can help reduce local and distant recurrence [9]. Care must be taken intraoperatively to avoid rupture of the tumour as the risk of local seeding is high [10,11]. The management of the recurrent laryngeal nerve is controversial. It should only be resected if there is evidence of tumour involvement or if it is not functioning preoperatively [12].

A decline in serum calcium should be expected in the first 24-hour postoperative period. The half-life of PTH is so short that the serum PTH level falls within minutes of successful surgery. Our patient's PTH level fell from 1743 ng/L preoperatively to 59.3 ng/L in the immediate postoperative period. The clinical course of this particular malignancy can be highly variable. Persistent or recurrent disease can occur in up to 50% of cases, while some patients are completely disease-free after the initial surgery. This contributes to the importance that should be placed on regular follow-up. In the literature, it seems apparent that tumour size does affect prognosis. The management of recurrent or metastatic parathyroid carcinoma reflects the rather indolent and surgical biology of this cancer compared with many other tumours [13].

## Conclusions

The prognosis of parathyroid carcinoma is quite variable. Not one characteristic correlates predictably with the outcome of prognosis. Early recognition and complete resection at the time of the initial surgery carry the best prognosis. The average time between surgery and the first recurrence is approximately three years, although intervals of up to 20 years have been reported. Once the tumour has recurred, complete cure is unlikely. A survey by the National Cancer Database reported 10-year survival rate to be approximately 49% [14].

## Consent

Written informed consent was obtained from the patient for publication of this case report and any accompanying images. A copy of the written consent is available for review by the Editor-in-Chief of this journal.

## Competing interests

The authors declare that they have no competing interests.

## Authors' contributions

KR drafted the article, performed the literature search, compiled the data, and acquired the images cited in this case report. NF assisted in performing the surgery and reviewed the manuscript. DOM supervised and edited the manuscript. EMD performed the surgery. All authors read and approved the final manuscript.
